# Potential of vegetation indices combined with laser-induced fluorescence parameters for monitoring leaf nitrogen content in paddy rice

**DOI:** 10.1371/journal.pone.0191068

**Published:** 2018-01-17

**Authors:** Jian Yang, Lin Du, Wei Gong, Shuo Shi, Jia Sun, Biwu Chen

**Affiliations:** 1 Faculty of Information Engineering, China University of Geosciences, Wuhan, Hubei, China; 2 State Key Laboratory of Information Engineering in Surveying, Mapping and Remote Sensing, Wuhan University, Wuhan, Hubei, China; 3 Collaborative Innovation Center of Geospatial Technology, Wuhan, Hubei, China; Kyung Hee Univeristy, REPUBLIC OF KOREA

## Abstract

Nitrogen (N) is important for the growth of crops. Leaf nitrogen content (LNC) serves as a crucial indicator of the growth status of crops and can help determine the dose of N fertilizer. Laser-induced fluorescence (LIF) technology and the reflectance spectra of crops are widely used to detect the biochemical content of leaves. Many vegetation indices (VIs) and fluorescence parameters have been developed to estimate LNC. However, the comparison among VIs and between fluorescence parameters and VIs has been rarely studied in the estimation of LNC. In this study, the performances of several published empirical VIs and fluorescence parameters for the estimation of paddy rice LNC were analyzed using the support vector machine (SVM) algorithm. Then, the optimal VIs (TVI, MTVI1, MTVI2, and MSAVI) and fluorescence parameters (F735/F460 and F685/F460), which were suitable for LNC monitoring in this study, were chosen. In addition, the combination of the VIs and fluorescence parameters was proposed as the input variables in the SVM model and used to estimate the LNC. Experimental results exhibited the promising potential of the LIF technology combined with reflectance for the accurate estimation of LNC, which provided guidance for monitoring the LNC.

## Introduction

Paddy rice is an important crop and a daily necessity to one-third of the world population. In China, approximately 30 million hectares of farming land are utilized each year to cultivate paddy rice. China is the leading producer of paddy rice in the world [1, 2]. Numerous studies have indicated that nitrogen (N) is a major nutrient element in crops and closely related to cereal crop yield [3–5]. To improve crop yield, excessive amounts of N fertilizers have been consumed and resulted in serious environmental problems. Thus, monitoring the N status of crops accurately will not only reduce the application amount of N fertilizers but also guarantee the quality of crops. Related studies have demonstrated that leaf nitrogen content (LNC) is a crucial indicator for estimating the dose of N level in crops. Numerous passive and active remote sensing technologies have been utilized to monitor LNC in cereal crops [6–9].

In passive remote sensing, several research groups reported that reflectance spectra are closely related to LNC [10] in that LNC can be determined by using reflectance spectra in the visible and near infrared regions of a leaf or canopy. Numerous vegetation indices (VIs) have been proposed to establish the correlation between VIs and LNC and used to estimate the latter. Several studies documented the high correlation of VIs (measured by various instruments) and LNC [11–13]. In addition, light detection and ranging (LiDAR) has also been successfully applied in the field of remote sensing as an active sensor. This technology can measure the three-dimensional information of the target surface and is useful in investigating certain biochemical properties [14–16]. The backscattered intensity of echoes is valuable in retrieving plant leaf chlorophyll content [17], LNC [18], and leaf water content[19]. Passive and active remote sensing technologies have been maturely utilized in satellite and airborne platforms and provide useful information for agricultural production and vegetation monitoring [20].

Laser-induced chlorophyll fluorescence, which differs from the reflectance spectra of remote sensing and LiDAR, was proposed by Chappelle et al. [21] to monitor crop growth status. Related studies have demonstrated that various nutrient stresses can be obtained through chlorophyll content monitoring. In addition, chlorophyll is a typical fluorophore in leaves, where chlorophyll fluorescence is widely applied to detect the photosynthetic activity of plants and monitor the effect of various nutrient stress factors on chlorophyll content [8, 22]. LNC monitoring has been investigated using different chlorophyll fluorescence parameters. The results displayed its advantages of rapidity, non-destructiveness, and high sensitivity [8, 23–25].

Several VIs based on reflectance spectra and fluorescence parameters based on fluorescence spectra have been designed to estimate LNC in crops on a leaf or canopy scale. However, the performances of published empirical VIs and fluorescence parameters have rarely been systematically tested in monitoring LNC in paddy rice. Comparisons between VIs and fluorescence parameters in terms of their estimation of LNC on a leaf scale are still rare. In addition, few studies have investigated the estimation of paddy rice LNC on the basis of the combination of VIs and fluorescence parameters. Therefore, the present study aims to (1) systematically analyze the performance of published empirical VIs and fluorescence parameters in the estimation of LNC using the support vector machine (SVM) algorithm, (2) compare the performances of VIs and fluorescence parameters in monitoring LNC, and (3) discuss the effectiveness of the proposed combination of VIs and fluorescence parameters in improving the monitoring accuracy of LNC.

## Materials and methods

### Ethics statement

Permission to access private lands, on which several sites were located, was obtained from landowners. Species surveys were conducted in accordance with the laws of the People’s Republic of China.

### Materials and experimental design

Yongyou 4949 of the three-line indica/japonica hybrid rice and Yangliangyou 6 hybrid indica rice were planted on April 27, 2014 and April 30, 2015, in experimental stations established in Junchuan County, Suizhou City, and Huazhong Agricultural University (HAU) in Wuhan City, China, respectively. During the entire growth period, six (0, 189, 229.5, 270, 310.5, and 351 kg/ha) and four (0, 120, 180, and 240 kg/ha) different doses of N fertilization of urea were utilized in 2014 and 2015, respectively. The most optimal doses of N fertilization were 270 and 180 kg/ha in 2014 and 2015, respectively, in accordance with the advice of the local farm extension service. N fertilization was divided into four splits (30% at seeding, 20% at tillering, 25% at shooting and 25% at booting) in 2014 and into three splits (60% at seeding, 20% at tillering and 20% at shooting) in 2015. The experimental field had a block design with three replications for each treatment under the same cultivation conditions [26]. The leaves of paddy rice were gathered on July 15, 2014 and July 26, 2015, which corresponded to the tillering stage of rice.

### Measurements of laser-induced fluorescence

The LIF system was built in a laboratory and consisted of three parts, namely, an excitation light source, optical receiver assembly and data collection system, and treatment part. The excitation light source is a neodymium-doped yttrium aluminum garnet laser and a third-harmonic generation. The emitted wavelength was 355 nm, and the width per pulse and output power were 5 ns and 1.5 mJ, respectively. The excitation light was transmitted perpendicular to the targets after passing several completely reflecting mirrors. To collect the back-emission fluorescence signal, a Maksutov-Cassegrain telescope was utilized in this system. An additional long-pass filter (Semrock BLP01-355R-25 with edge of 361 nm and 93% transmittance at 364.9–900 nm) was placed behind the telescope and used to eliminate the reflected light from the laser entering the optical fiber. Then, a single-mode optical fiber with a diameter of 200 μm was utilized to transmit the fluorescence collected between the telescope and spectrograph (Princeton Instrument SP2500i with spectral resolution of 0.5 nm). An intensified charge coupled device camera was utilized to detect the excited fluorescence signals passing through the spectrograph. A personal computer was utilized to store and post-process these fluorescence data conveniently. In this study, the fluorescence spectra ranged from 360 nm to 800 nm, and the sampling interval was 0.5 nm. To eliminate the oscillation of the excitation light, each leaf sample was measured five times to calculate the average fluorescence spectral curve for each sample.

### Acquisition of leaf reflectance spectra

In this study, leaf reflectance spectra were obtained using an ASD FieldSpec Pro FR (Analytical Spectral Devices, Inc., Boulder, USA) which is a commercial passive instrument. The spectral acquisition process was conducted following the study of Pu et al. [27]. A 100 W halogen reflectorized lamp served as the light source. Each paddy rice leaf sample was measured thrice to acquire an average reflectance spectrum for each sample at the same position where the fluorescence was measured. All reflectance spectra were obtained at the nadir direction of the radiometer, and the field angle of the receiving optical fiber was 25°. The distance between the leaf sample and the optical fiber probe was approximately 4 cm. The entire reflectance spectral radiance changed from 350 nm to 2500 nm with a 1 nm spectral resolution. A reference standard whiteboard (Spectralon, Labsphere, Inc., North Sutton, NH, USA, 10 cm × 10 cm, reflectance nearly 99%) was utilized as the reference for converting the raw leaf radiance to spectral reflectance. The whiteboard was measured every 10 min during the entire leaf radiance measurement procedure [28]. The leaf reflectance spectrum could be obtained as follows:

$$R_{\lambda }=R_{L}(\lambda )/R_{R}(\lambda )$$ (1)

where *R*_*L*_(*λ*) and *R*_*R*_(*λ*) represent the leaf and reference standard white board radiances at wavelength *λ*, respectively.

### Measurement of leaf nitrogen content

Leaves were destructively sampled by randomly cutting six fully expanded the second leaves from the top with three replicates in each experimental field. These paddy rice leaves were sealed in plastic bags, stored in an ice chest, and immediately transported to the laboratory for reflectance and fluorescence spectral measurements [28]. All samples were immediately sent to Wuhan Academy of Agricultural Science and Technology for the confirmation of LNC after spectral measurements. The traditional Kjeldahl method was utilized to measure the LNC [29].

### VIs of spectral reflectance and fluorescence parameters

In this study, 67 published empirical VIs (formula and detailed description in Appendix) [6, 11, 16, 18, 30–34] were used to analyze the paddy rice LNC measured by ASD. All VIs could be utilized in this investigation because the reflectance spectral range was 350 nm to 2500 nm. The spectral resolution was 1 nm, and the corresponding spectral reflectance was utilized to calculate these VIs. For the fluorescence spectra, seven published empirical fluorescence parameters (F_740_/F_685_, F_740_/F_460_, F_685_/F_460_, F_685_/F_525_, F_740_/F_525_, (F_460_-F_685_)/(F_460_+F_685_), and (F_460_-F_740_)/(F_460_+F_740_). F_740_, F_685_, F_525_, and F_460_ denoted the intensity of fluorescence at 740, 685, 525, and 460 nm, respectively) [23, 35, 36] were employed to estimate the LNC through LIF.

### Analytical method

SVM, which is a classical supervised learning algorithm that has the capacity to construct linear and nonlinear inversions, was implemented in this investigation. In comparison with the artificial neural network, SVM has a strong theoretical foundation in statistical theory and exhibits remarkable performance (accuracy on test sets) in practice. Furthermore, SVM is insensitive to the dimension number of training samples and requires a small number of training samples. Detailed description of SVM can be found in the references [37, 38]. In addition, the kernel function is a crucial part of SVM analysis. According to related studies [39], the analysis of variance kernel, which is a radial basis function kernel and just as the Gaussian and Laplacian kernels, was utilized as a Kernel function of SVM and could be written as follows:

$$K(X_{i},Y_{i})={\left(\sum_{i}\exp(-\gamma {\left(X_{i}-Y_{i}\right)}^{2})\right)}^{d}$$ (2)

where *γ* denotes a kernel parameter, *Y*_*i*_ represents the training output, *X*_*i*_ stands for the training inputs, and *d* is a constant.

Wavelet transform is similar to Fourier transform but uses a completely different merit function. The capability of wavelets to provide multiresolution low entropy makes them an ideal tool for studying spectra [40]. Before analysis, the spectra were denoised and smoothened through wavelet transform [41]. The fluorescence parameters and VIs were calculated, and these measured datasets were then randomly divided into four equal parts. Four-fold cross validation was utilized to analyze the performance of these parameters in the estimation of LNC. Three-fourths of the data were utilized to train the SVM model, and the remaining quarter was utilized in the testing. This procedure was conducted four times, utilizing a different quarter of data as the test sets each time. The coefficient of determination (*R*^2^), root mean square error (*RMSE*), and relative error (*RE*) in the prediction were utilized to discuss the performance of the model on the basis of different spectral characteristics. *RMSE* and *RE* can be written as follows:

$$RMSE=\sqrt{\frac{1}{n}\sum_{i=1}^{n}{\left(X_{p,i}-X_{o,i}\right)}^{2}}$$ (3)

$$RE=\frac{100}{{\bar{X}}_{o}}RMSE$$ (4)

Where *n* denotes the number of samples, *X*_*p*,*i*_ represents the predicted values, *X*_*o*,*i*_ corresponds to the measured values, and ${\bar{X}}_{o}$ represents the mean of the measured values. Low *RMSE* and *RE* and high *R*^2^ indicate a high accuracy and precision of a model in predicting LNC [26, 42].

## Results

### Fluorescence and reflectance spectra

**Fig 1:**
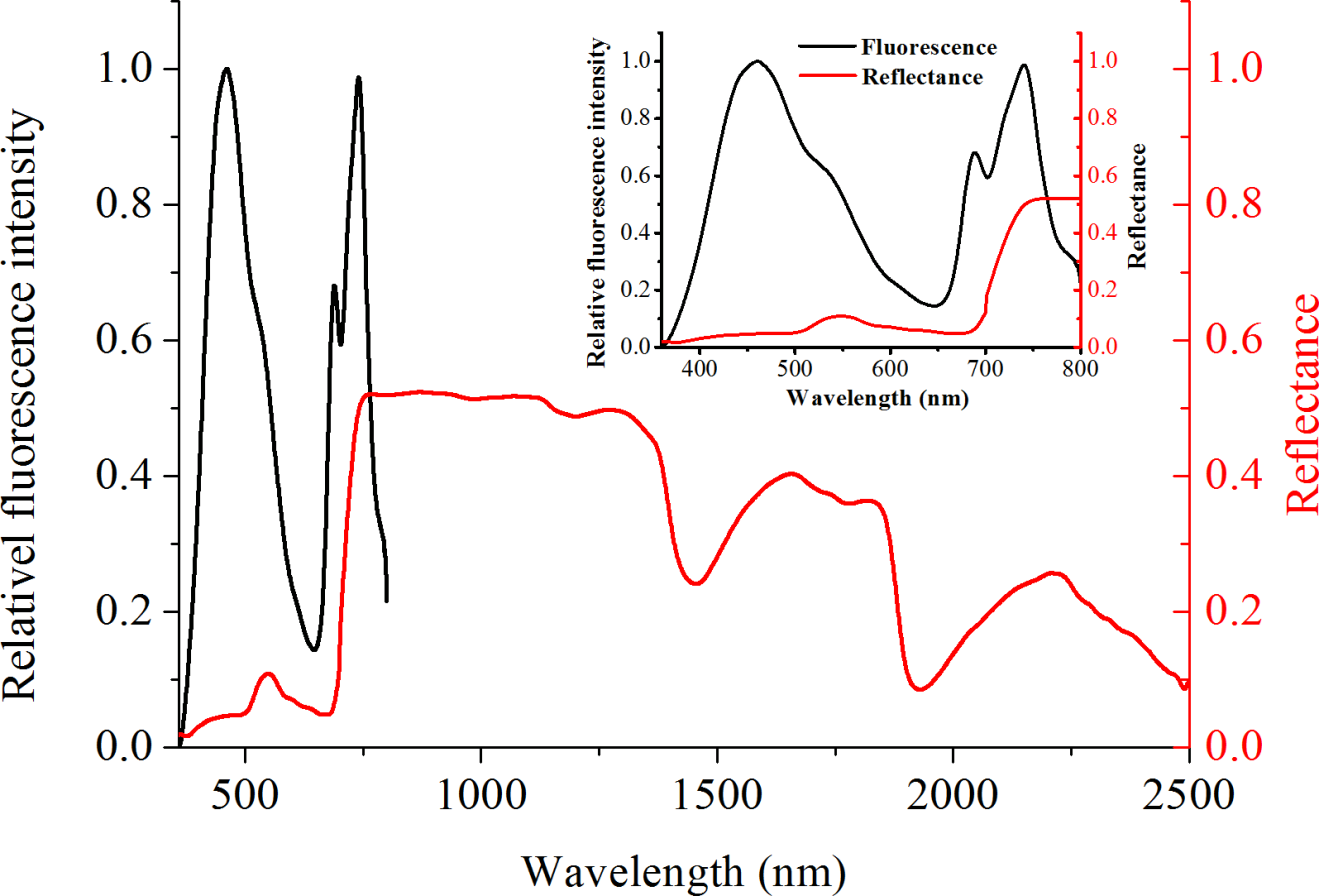
Change in fluorescence intensity (measured by LIF system) and leaf reflectance (measured by ASD) with wavelength. The black and red solid lines denote the fluorescence and reflectance spectra, respectively. The minimal graph exhibits the same wavelength range for the fluorescence and reflectance spectra.

Fig 1 shows the completely different spectral characteristics exhibited by the fluorescence and reflectance spectra.

The fluorescence spectra ranged from 360 nm to 800 nm and exhibited three main fluorescence peaks at 440–465 nm, 680–690 nm, and 730–740 nm. According to previous studies [43, 44], the center wavelengths of the three fluorescence peaks were 460, 685, and 735 nm. Then, published empirical fluorescence parameters calculated by these fluorescence peaks were utilized to analyze LNC.

The red solid line shown in Fig 1 is the typical plant reflectance. The reflectance spectrum has a wider spectral range than the fluorescence spectrum. However, the spectral information, which is closely related to LNC, was limited. LNC was assessed using the reflective spectral characteristics, which is a method that has been proven by numerous researchers. A total of 67 published empirical VIs were calculated in this study by utilizing the acquired reflectance through ASD.

### Relationship of VIs and fluorescence parameters with LNC

**Fig 2:**
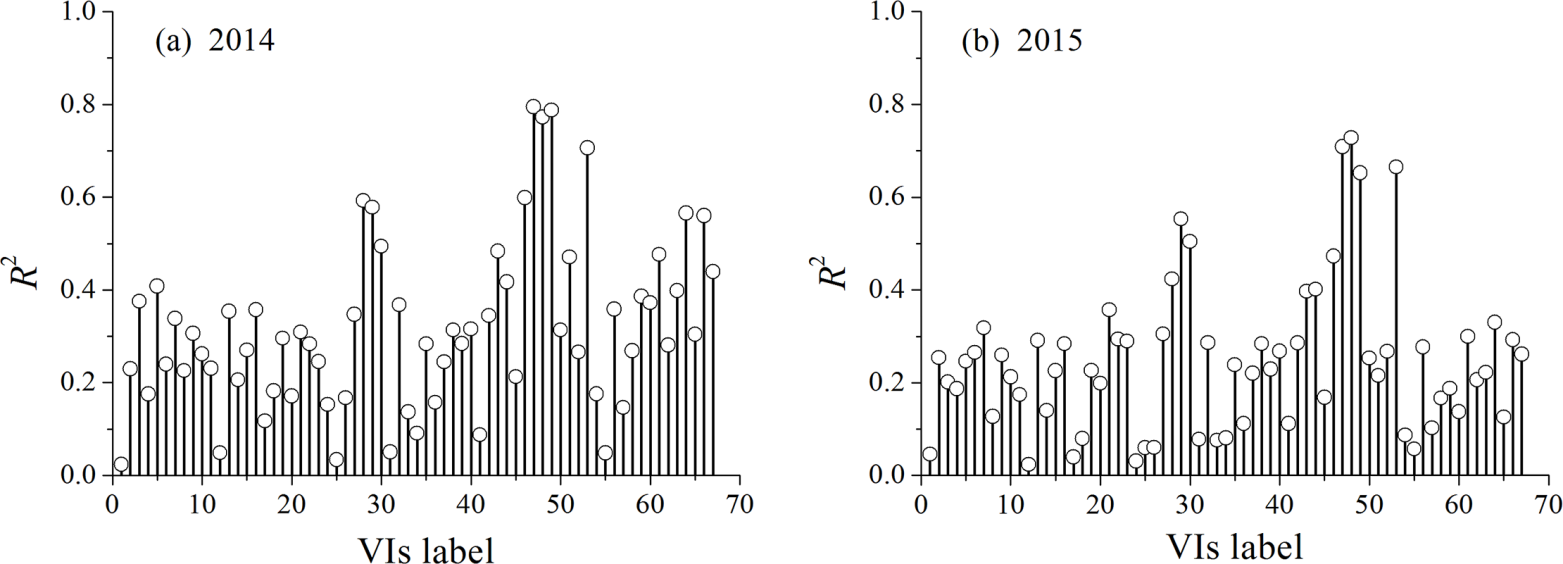
Coefficient of determination (*R*^2^) between different vegetation indices (VIs) based on the reflectance spectra and leaf nitrogen content (LNC) in growing years (a) 2014 and (b) 2015.

Fig 2 shows the correlation between each of the VIs and LNC.

As shown in Fig 2, the different VIs exhibited varying correlations. The experimental results demonstrated that TVI, MTVI1, MTVI2, and MSAVI displayed higher correlations (*R*^2^ values were 0.79, 0.77, 0.78, and 0.70 for 2014 and 0.72, 0.73, 0.65, and 0.66 for 2015) than the other VIs. The correlation between the fluorescence parameters and LNC was investigated in our previous study [45]. The investigation demonstrated that fluorescence ratios F735/F460 and F685/F460 were closely related to LNC in paddy rice [46].

### Performance of VIs and fluorescence parameters in estimating LNC

**Fig 3:**
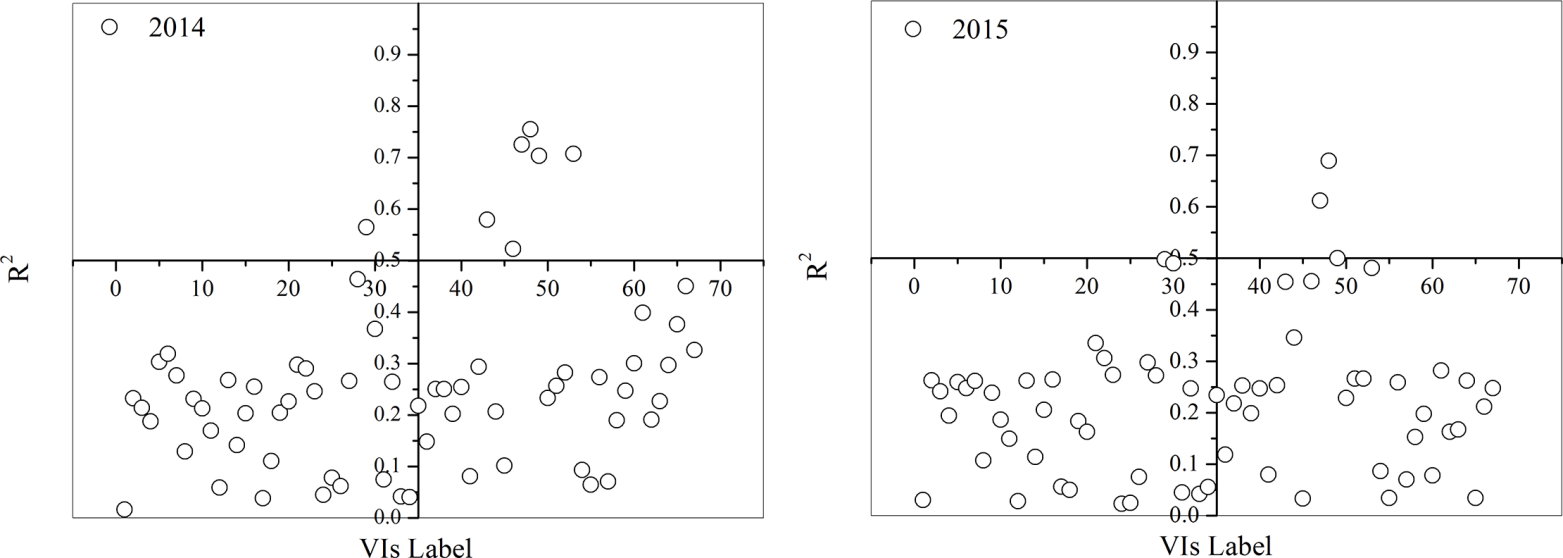
Coefficient of determination (*R*^2^) between predicted LNC using single vegetation indices (VIs) obtained through SVM model and measured LNC in growing years (a) 2014 and (b) 2015.

To analyze the performances of all VIs and fluorescence parameters in estimating LNC, each of the parameters was used as a single input variable to predict LNC through SVM. Fig 3 presents the *R*^2^ of the linear regression of predicted and measured LNC.

Fig 3 shows the performance of single VIs in monitoring LNC in paddy rice. The experimental results indicated that TVI, MTVI1, MTVI2, and MSAVI were superior to the other VIs with higher *R*^2^ values for the two growing years (2014 and 2015). A detailed analysis of the performance of fluorescence parameters in estimating LNC in paddy rice is found in reference [45]. In our previous investigation, the results demonstrated that the F735/F460 and F685/F460 were superior to the other fluorescence parameters in estimating LNC. Then, the combination of the four VIs and the two fluorescence parameters was proposed to estimate LNC.

### Estimation of LNC using SVM model

**Fig 4:**
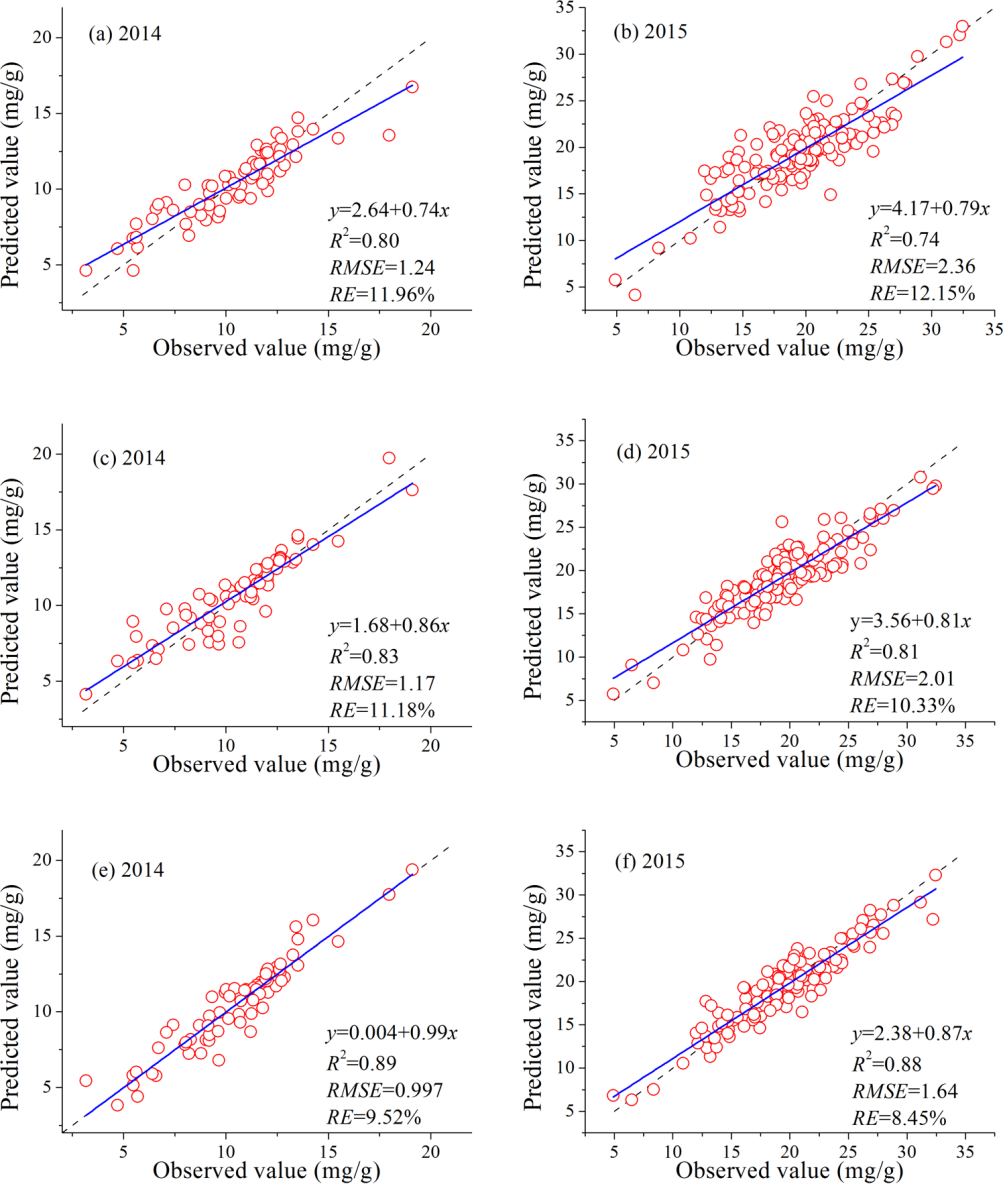
**Relationship between predicted and measured LNC using different types of characteristic parameters** ((a) and (b): four VIs; (c) and (d): two fluorescence parameters; and (e) and (f): four VIs combined with two fluorescence parameters) from different growth years and calculated using SVM. (a), (c), and (e): 2014; (b), (d), and (f): 2015. The dotted line denotes the 1:1 line; the blue solid line represents the fitted curves for the regression model.

To compare the performances of the three different types of characteristics parameters (VIs, fluorescence parameters, and the combination of VIs and fluorescence parameters), SVM was utilized to estimate LNC. The measured dataset was automatically divided into four equal parts, and four-fold cross validation was used. Fig 4 presents the relationships between predicted and measured LNC.

Fig 4 shows the relationship between observed LNC and that predicted using SVM with different characteristic parameters for growth years 2014 and 2015. The blue solid lines show the linear regression between the predicted and observed values, whereas the dotted lines represent the 1:1 line. Ideally, the blue solid line should coincide with the 1:1 line. By comparing these results and identifying high *R*^2^ and low *RMSE* and *RE*, Fig 4 demonstrates that fluorescence parameters (*R*^2^ = 0.83 and 0.81 for 2014 and 2015, respectively) were superior to the VIs (*R*^2^ = 0.80 and 0.74 for 2014 and 2015, respectively). In addition, the combination of VIs and fluorescence parameters provided higher accuracy in LNC monitoring than either fluorescence parameters or VIs alone. The *R*^2^ of the regression analysis between the predicted and measured LNC reached 0.89 and 0.88 for 2014 and 2015, respectively.

## Discussion

At present, the amount of VIs based on reflectance and LIF spectral data has already been utilized to analyze the growth status of vegetation, but only a few studies have compared VIs and fluorescence parameters in term of estimating LNC in paddy rice. [30, 47, 48]. In this investigation, published empirical VIs and fluorescence parameters were utilized to monitor LNC. The experimental results demonstrated that TVI, MTVI1, MTVI2, and MSAVI had higher *R*^2^ and lower *RMSE* and *RE* than other VIs and therefore were suitable for estimating LNC in paddy rice [49, 50]. The spectral bands of these VIs are 550 nm, 670 nm, 750 nm, and 800 nm. These bands were good indicator bands for LNC detection, as demonstrated in relative studies [30]. In addition, LNC can be influenced by the leaf area index of vegetation, and the latter may be the main factor that controls the reflectance spectra in the visible and near-infrared regions [32]. Haboudane et al. concluded that these VIs were developed to eliminate the effects of interference factors on the detection of vegetation [50].

In this study, fluorescence spectra exhibited three main fluorescence peaks at 440–465 nm, 680–690 nm, and 730–740 nm. According to previous studies [43, 44], the fluorescence peak at 460 nm was attributed to nicotinamide adenine dinucleotide, whereas those at 685 and 735 nm were responsible for the chlorophyll a of Photosystem II and antenna chlorophyll of photosystems I and II, respectively. Related studies have demonstrated that LNC is closely related to the fluorescence peaks (685 and 735 nm) [36, 51]. The current research demonstrated that fluorescence parameters (*R*^2^ = 0.83, 0.81 for 2014 and 2015, respectively) were superior to VIs (*R*^2^ = 0.80 and 0.74 for 2014 and 2015, respectively) in the estimation of LNC. The probable interpretation is that the fluorescence parameters were influenced by the re-absorption of leaf internal fluorophore and were relatively unsusceptible to ambient factors in comparison with the VIs [52, 53]. Meanwhile, LIF technology is widely implemented in the detection of crop physiological property and a popular topic in the field of remote sensing [8, 24, 45]. Malenovsky et al. confirmed that the chlorophyll content in leaves degrade and decrease rapidly, and lutein then turns into a major pigment component when LNC in the leaf is reduced to threshold levels, thereby in turn affect the fluorescence characteristics of leaves [46]. The proposed combination of VIs with fluorescence parameters provided a more accurate monitoring of LNC in comparison with either fluorescence parameters or VIs alone through the SVM model (*R*^2^ = 0.89 and 0.88 for 2014 and 2015, respectively) [54]. The satisfactory results of the SVM model based on the combination of VIs and fluorescence parameters indicated their promising potential in the monitoring of LNC in paddy rice.

In this investigation, the performances of VIs and fluorescence parameters in estimating LNC were compared in detail. In addition, the combination of VIs with fluorescence parameters was proposed to estimate LNC in paddy rice. However, this preliminary investigation only compared the VIs and fluorescence parameters in terms of monitoring LNC in paddy rice using the SVM model. Certain limitations should be considered in future studies. For the SVM algorithm, the kernel function is a key factor, and the effect of different kernel functions on LNC monitoring needs to be discussed in future work. Furthermore, to obtain a solid conclusion and improve the generalization capability of the proposed approach, additional paddy rice cultivars, growth seasons, and other crops should be considered in future studies.

## Conclusion

This research investigated the performances of VIs and fluorescence parameters ub the estimation of LNC in paddy rice. The experimental results demonstrated that TVI, MTVI1, MTVI2, and MSAVI were superior to the other VIs in monitoring LNC. In addition, the comparison of the VIs and fluorescence parameters for the estimation of LNC through SVM demonstrated that the fluorescence parameters (*R*^2^ = 0.83 and 0.81 in 2014 and 2015, respectively) were superior to the VIs (*R*^2^ = 0.80 and 0.74 in 2014 and 2015, respectively). Finally, the combination of VIs and fluorescence parameters was proposed to estimate LNC. The experimental results demonstrated that the proposed combination could effectively improve the accuracy of LNC estimation (*R*^2^ = 0.89 and 0.88 in 2014 and 2015, respectively). Thus, the LNC in paddy rice could be accurately evaluated by implementing the LIF technology combined with hyperspectral reflectance. Nevertheless, further studies using additional crops cultivars and growth years are still required to obtain a solid conclusion and improve the generalization capability of the proposed approach.

## Appendix

### Formula of vegetation indices

Vegetation indices (VIs) based on reflectance spectra and the corresponding calculation formula in this paper [6, 11, 16, 18, 30–34].

Normalized difference vegetation index:

$$\mathrm{NDVI}1=\left(R_{800}-R_{670}\right)/\left(R_{800}+R_{670}\right)$$ (1)

$$\mathrm{NDVI}2=\left(R_{780}-R_{670}\right)/\left(R_{780}+R_{670}\right)$$ (2)

$$\mathrm{NDVI}3=\left(R_{573}-R_{440}\right)/\left(R_{573}+R_{440}\right)$$ (3)

$$\mathrm{NDVI}4=\left(R_{410}-R_{365}\right)/\left(R_{410}+R_{365}\right)$$ (4)

$$\mathrm{NDVI}5=\left(R_{503}-R_{483}\right)/\left(R_{503}+R_{483}\right)$$ (5)

$$\mathrm{NDVI}6=\left(R_{800}-R_{680}\right)/\left(R_{800}+R_{680}\right)$$ (6)

$$\mathrm{NDVI}7=\left(R_{1220}-R_{710}\right)/\left(R_{1220}+R_{710}\right)$$ (7)

$$\mathrm{NDVI}8=\left(R_{801}-R_{550}\right)/\left(R_{801}+R_{550}\right)$$ (8)

Normalized difference index:

$$\mathrm{NDI}1=\left(R_{790}-R_{720}\right)/\left(R_{790}+R_{720}\right)$$ (9)

$$\mathrm{NDI}2=\left(R_{860}-R_{720}\right)/\left(R_{860}+R_{720}\right)$$ (10)

$$\mathrm{NDI}3=\left(R_{750}-R_{705}\right)/\left(R_{750}+R_{705}\right)$$ (11)

$$\mathrm{NDI}4=\left(R_{570}-R_{531}\right)/\left(R_{570}+R_{531}\right)$$ (12)

$$\mathrm{NDI}5=\left(R_{780}-R_{710}\right)/\left(R_{780}-R_{680}\right)$$ (13)

$$\mathrm{NDI}6=\left(R_{850}-R_{710}\right)/\left(R_{850}-R_{680}\right)$$ (14)

$$\mathrm{NDI}7=\left(R_{734}-R_{747}\right)/\left(R_{715}+R_{726}\right)$$ (15)

$$\mathrm{mNDI}=\left(R_{750}-R_{705}\right)/\left(R_{750}+R_{705}-2\times R_{445}\right)$$ (16)

Simple ratio vegetation index

$$\mathrm{SR}1=R_{700}/R_{670}$$ (17)

$$\mathrm{SR}2=R_{750}/R_{550}$$ (18)

$$\mathrm{SR}3=R_{750}/R_{700}$$ (19)

$$\mathrm{SR}4=R_{780}/R_{670}$$ (20)

$$\mathrm{SR}5=R_{787}/R_{765}$$ (21)

$$\mathrm{SR}6=R_{553}/R_{537}$$ (22)

$$\mathrm{SR}7=R_{545}/R_{538}$$ (23)

$$\mathrm{SR}8=R_{554}/R_{677}$$ (24)

$$\mathrm{SR}9=R_{801}/R_{670}$$ (25)

$$\mathrm{SR}10=R_{800}/R_{550}$$ (26)

$$\mathrm{SR}11=R_{740}/R_{720}$$ (27)

$$\mathrm{SR}12=R_{670}/\left(R_{700}•R_{650}\right)$$ (28)

$$\mathrm{SR}13=R_{672}/\left(R_{708}•R_{550}\right)$$ (29)

$$\mathrm{SR}14=R_{860}/\left(R_{708}•R_{550}\right)$$ (30)

$$\mathrm{PSSRa}=R_{800}/R_{680}$$ (31)

$$\mathrm{PSSRb}=R_{800}/R_{635}$$ (32)

$$\mathrm{SR}15=R_{750}/R_{705}$$ (33)

$$\mathrm{SR}16=R_{950}/R_{660}$$ (34)

$$\mathrm{SR}17=R_{990}/R_{720}$$ (35)

$$\mathrm{SR}18=R_{780}/R_{740}$$ (36)

$$\mathrm{SR}19=R_{743}^{\prime }/R_{1316}^{\prime }$$ (37)

$$\mathrm{SR}20=R_{730}^{\prime }/R_{705}^{\prime }$$ (38)

Zarco-Tejada&Miller:

$$\mathrm{ZTM}=R_{760}/R_{710}$$ (39)

Optimized vegetation index

$$\mathrm{VIopt}2=R_{760}/R_{730}$$ (40)

$$\mathrm{MSR}1=\left(R_{800}/R_{670}-1\right)/\sqrt{R_{800}/R_{670}+1}$$ (41)

$$\mathrm{MSR}2=\left(R_{750}/R_{705}-1\right)/\sqrt{R_{750}/R_{705}+1}$$ (42)

Chlorophyll absorption ratio index:

$$\mathrm{CARI}=(R_{700}-R_{670})-0.2\times (R_{700}+R_{550})$$ (43)

Modified chlorophyll absorption ratio index:

$$\mathrm{MCARI}1=[(R_{700}-R_{670})-0.2\times (R_{700}-R_{550})]•(R_{700}/R_{670})$$ (44)

$$\mathrm{MCARI}2=[(R_{750}-R_{705})-0.2\times (R_{750}-R_{550})]•(R_{750}/R_{705})$$ (45)

Transformed chlorophyll absorption ratio index:

$$\mathrm{TCARI}=3\times [(R_{700}-R_{670})-0.2\times (R_{700}-R_{550})(R_{700}/R_{670})]$$ (46)

Triangular vegetation index:

$$\mathrm{TVI}=0.5\times [120\times (R_{750}-R_{550})-200\times (R_{670}-R_{550})]$$ (47)

Modified Triangular vegetation index:

$$\mathrm{MTVI}1=1.2\times [1.2\times (R_{800}-R_{550})-2.5\times (R_{670}-R_{550})]$$ (48)

$$\mathrm{MTVI}2=\frac{1.5\times [1.2\times (R_{800}-R_{550})-2.5\times (R_{670}-R_{550})]}{\sqrt{{\left(2\times R_{800}+1\right)}^{2}-(6\times R_{800}-5\times \sqrt{R_{670}})-0.5}}$$ (49)

Red edge position linear:

$$\mathrm{REP}=710+50\times \frac{0.5\times (R_{810}+R_{660})-R_{710}}{R_{760}+R_{710}}$$ (50)

Optimized soil adjusted vegetation index:

$$\mathrm{OSAVI}1=(1+0.16)\times \frac{R_{800}-R_{670}}{R_{700}+R_{670}+0.16}$$ (51)

$$\mathrm{OSAVI}2=(1+0.16)\times \frac{R_{750}-R_{705}}{R_{750}+R_{705}+0.16}$$ (52)

$$\mathrm{MSAVI}=0.5\times [2\times R_{800}+1-\sqrt{{\left(2\times R_{800}+1\right)}^{2}-8\times (R_{800}-R_{670})}]$$ (53)

$$\mathrm{TBI}1=R_{705}/\left(R_{717}+R_{491}\right)$$ (54)

$$\mathrm{TBI}2=R_{1310}/\left(R_{1720}+R_{730}\right)$$ (55)

$$\mathrm{TBI}3=\left(R_{924}-R_{703}+2R_{423}\right)/\left(R_{924}+R_{703}-2R_{423}\right)$$ (56)

Red edge model index:

$$\mathrm{R\_M}=R_{750}/R_{720}-1$$ (57)

Green model index:

$$\mathrm{G\_M}=R_{750}/R_{550}-1$$ (58)

$$\mathrm{PNC}=\exp[2.5-23.5\times \frac{R_{503}-R_{483}}{R_{503}+R_{483}}]$$ (59)

$${{\mathrm{TCARI}}_{i}/\mathrm{OSAVI}}_{j}\;\left(j=1,\;2\right)$$ (60)

$$\mathrm{CARI}/{\mathrm{OSAVI}}_{j}\;\left(j=1,\;2\right)$$ (61)

$${\mathrm{MCARI}}_{i}/{\mathrm{OSAVI}}_{j}\;\left(i=1,\;2;j=1,\;2\right)$$ (62)
